# Plasma NT-proBNP and white matter hyperintensities in type 2 diabetic patients

**DOI:** 10.1186/1475-2840-11-119

**Published:** 2012-10-03

**Authors:** Henrik Reinhard, Ellen Garde, Arnold Skimminge, Per Åkeson, Thomas Zoëga Ramsøy, Kaj Winther, Hans-Henrik Parving, Peter Rossing, Peter K Jacobsen

**Affiliations:** 1 Steno Diabetes Center, Gentofte, Denmark; 2 Danish Research Centre for Magnetic Resonance, Copenhagen University Hospital, Hvidovre, Denmark; 3 Department of Clinical Biochemistry, Frederiksberg University Hospital, Frederiksberg, Denmark; 4 Department of Medical Endocrinology, University Hospital of Copenhagen, Copenhagen, Denmark; 5 Faculty of Health Science, Aarhus University, Aarhus, Denmark; 6 The Heart Centre, Rigshospitalet & University of Copenhagen, Copenhagen, Denmark; 7 Decision Neuroscience Research Group, Dept. of Marketing, Copenhagen Business School, Frederiksberg, Denmark

**Keywords:** Type 2 diabetes, Plasma NT-proBNP, 3-D magnetic resonance imaging, White matter hyperintensities, Brain parenchymal fraction

## Abstract

**Abstract:**

Elevated plasma N-terminal (NT)-proBNP from the heart as well as white matter hyperintensities (WMH) in the brain predict cardiovascular (CV) mortality in the general population. The cause of poor prognosis associated with elevated P-NT-proBNP is not known but WMH precede strokes in high risk populations. We assessed the association between P-NT-proBNP and WMH or brain atrophy measured with magnetic resonance imaging (MRI) in type 2 diabetic patients, and age-matched controls.

**Methods and results:**

We measured P-NT-proBNP(ng/l) in 20 diabetic patients without prior stroke but with(n = 10) or without(n = 10) asymptomatic coronary artery disease(CAD) in order to include patients with a wide-ranging CV risk profile. All patients and 26 controls had a 3D MRI and brain volumes(ml) with WMH and brain parenchymal fraction(BPF), an indicator of brain atrophy, were determined.

P-NT-proBNP was associated with WMH in linear regression analysis adjusted for CV risk factors(r = 0.94, p = 0.001) and with BPF in univariate analysis(r = 0.57, p = 0.009). Patients divided into groups of increased P-NT-proBNP levels were paralleled with increased WMH volumes(geometric mean[SD];(2.86[5.11] ml and 0.76[2.49] ml compared to patients with low P-NT-proBNP 0.20[2.28] ml, p = 0.003)) and also when adjusted for age, sex and presence of CAD(p = 0.017). The association was strengthened by CV risk factors and we did not find a common heart or brain specific driver of both P-NT-proBNP and WMH. Patients and particular patients with CAD had higher WMH, however no longer after adjustment for age and sex.

**Conclusion:**

P-NT-proBNP was associated with WMH in type 2 diabetic patients, suggesting a linkage between heart and brain disease.

## Introduction

Manifest atherosclerosis that includes strokes is the most important determinant of the excessive morbidity and mortality in patients with type 2 diabetes, especially in patients with microalbuminuria [1]. Although medical treatment aimed at reduction of established conventional risk factors is effective in reducing the increased cardiovascular morbidity and mortality in patients with diabetes, there is still excess morbidity and mortality compared to the background population [2]. Detection of subclinical atherosclerotic manifestations in patients with diabetes in order to be able to identify patients at risk and start appropriate intervention at an early stage therefore seems evident.

Elevated plasma N-terminal-proBNP (P-NT-proBNP) levels released from the heart as well as white matter hyperintensities (WMH) in the brain are risk factors for cardiovascular (CV) morbidity and mortality in the general population [3,4]. We have previously identified P-NT-proBNP as a powerful predictor of CV mortality that included strokes in patients with type 2 diabetes [5]. The cause of poor prognosis seen in patients with elevated P-NT-proBNP is not known, but P-NT-proBNP is associated with the diagnosis of stroke in patients with suspected acute cerebrovascular disease [6]. Furthermore, a recent study (25% had diabetes) showed that P-NT-proBNP is highly elevated in patients with acute and cardioemboli strokes compared to patients with other types of strokes [7]. WMH, defined as white matter areas with high signal intensities on T2-weighted magnetic resonance imaging(MRI) are regarded as expressions of chronic hypoperfusion [8]. Presence of WMH portends a three-fold increased risk of stroke and two-fold increased risk of dementia or mortality in the general population [4]. WMH are together with brain atrophy regarded as surrogate markers for cerebral small vessel disease. Cerebral small vessel disease is the most prevalent asymptomatic neurological disease, its incidence reported to be 6- to 10-fold that of symptomatic stroke [9,10]. Whether P-NT-proBNP is associated with WMH and/or brain atrophy in patients with type 2 diabetes, is not known. In this study, we assessed the association between P-NT-proBNP and WMH and/or brain atrophy measured with MRI in type 2 diabetic without prior history of stroke but with or without asymptomatic coronary artery disease (CAD), and age and sex matched controls.

## Methods

### Patient cohort and investigations

We have previously investigated the extent of asymptomatic coronary artery disease as defined by myocardial imaging (MPI) and/or coronary angiography (CAG) in a study of 200 patients with type 2 diabetes and microalbuminuria. The design and a selection of clinical measurements of the study have previously been described [11]. In the present study, we randomly selected 20 patients without previous clinical strokes but with or without previously screen detected asymptomatic CAD based on our previous study and in order to include patients with a wide-ranging CV risk profile. We also included 26 age and sex matched controls without known diabetes or CAD. P-NT-proBNP was analysed by an established immunoassay [5]. Tests for autonomic neuropathy, heart rate variability assessed by the expiration-inspiration variation of the heart rate and somatic nerve function (vibratory perception threshold) evaluated by biothesiometry, were performed.

The cardiovascular examinations have previously been described [11-13]. In brief, carotid artery intima media thickness (CIMT) was measured at the posterior wall approximately 20 mm proximal to the bifurcation bilaterally and calculated as the mean of CIMT on both sides. Agaston coronary calcium score (CCS) was measured during a single breath hold using a 16 multidetector-row CT scanner with 3 mm slice thickness. Transthoracic echocardiography was performed using a Philips IE 33 machine. Significant CAD was defined as the presence of significant myocardial perfusion defects on MPI, and/or >70% coronary artery stenosis at CAG. The correlations between P-NT-proBNP, CCS, MPI and CAG have previously been reported [11]. All subjects underwent brain MRI (see below) and patients were assessed with the Mini Mental Status Examination (MMSE), a screening tool widely used for impaired cognitive skills [14]. It is often used to identify individuals in the early phase of dementia.

### Brain magnetic resonance imaging

All subjects were scanned using a Siemens Magnetom Trio 3 T MRI scanner(Erlangen, Germany) with an eight channel head coil (Invivo, FL, USA) High-resolution three-dimensional(192 sagittal slices; 256 x 256 acquisition matrix) structural MRI scans of the brain were acquired including T1-weighted magnetisation prepared rapid acquisition gradient echo (MPRAGE) images (repetition time (TR) = 1550 ms, echo time (TE) = 3.04, inversion time(TI) = 800 ms; flip-angle = 9°; 1x1x1mm3), T2-weighted turbo spin echo images (TR = 3000 ms, TE = 354 ms; FOV = 282 mm;1.1x1.1x1.1 mm3), and fluid-attenuated inversion recovery (FLAIR) images (TR = 6000 ms, TE = 353 ms, TI = 2200; FOV = 282 mm;1.1x1.1x1.1 mm3).

### Image pre-processing

Images were pre-processed using pipelines implemented in Matlab, mainly SPM8 (Wellcome Department of Cognitive Neurology, University College London, UK) routines to obtain anatomical point correspondence between images. MPRAGE images were initially coregistered to the Monteal Neurological Institute (MNI) template image using a 6 parameter rigid transformation. Subsequently, T2-weighted and FLAIR images were coregistered to the corresponding MPRAGE image using a 6 parameter rigid transformation. All images were corrected for spatial distortions due to non-linearity in the gradient system of the scanner, and resliced to 1x1x1 mm3 resolution in MNI standard orientation.

### Global tissue volumes

Brain tissue volumes, that included cortex, grey and white matter were estimated from the MPRAGE image with SIENAX, part of FSL, and were normalised for subject head size [15]. Intracranial volume was calculated by multiplying the volumetric scaling factor with the intracranial volume of the standard MNI template. Brain parenchymal fraction (BPF), a surrogate for brain atrophy was determined as total brain volume/ICV.

### White matter hyperintensities

WMH were defined as clearly hyperintense areas relative to surrounding white matter on both FLAIR and T2-weighted images and identified by simultaneous inspection of both images. Infarcts (areas that were hyperintense on T2-weighted imaging but with low signal intensity on FLAIR and T1-weighted images and located in a vascular distribution area) were noted but not included. Local threshold was applied and WMH volumes for the whole brain quantified automatically using JIM (http://www.xinapse.com). Visual identification was carried out by a single trained rater blinded to clinical information.

The study was approved by the local ethics committee and all patients gave written informed consent.

### Statistical analysis

We investigated the association between P-NT-proBNP and WMH and/or BPF in univariate linear regression analyses in the 20 cases and also assessed the WMH levels in patients divided according to P-NT-proBNP tertiles. Furthermore, multivariate linear regression analyses were also performed with adjustment for age, sex and CV risk factors. Importantly, due to our low number of samples, we only included 2–3 risk factors in addition to age and sex for every analysis. However, we did test to see if various different combinations of various different 2–3 CV risk factors changed associations between P-NT-proBNP and WMH and/or BPF. Included CV risk factors were known diabetes duration, BMI, total cholesterol, systolic blood pressure, HbA1c, presence of retinopathy, urinary albumin excretion rate, vibration threshold, heart rate variation, CIMT, left ventricular ejection fraction (LVEF), CCS and subclinical CAD. Particular the concomitant analyses with measures of subclinical atherosclerosis (CIMT, LVEF, CCS and subclinical CAD) were performed in order to test for a possible common driver of both P-NT-proBNP and WMH. Secondly, patients in groups with or without CAD and age-matched controls were compared in relation to brain measurements. Comparisons between groups were performed by an unpaired Student's t-test or the Pearson Chi-test^2^ when appropriate. Data were expressed as means and standard deviation (SD), except for non-normally distributed variables, which were given as medians and interquartile range. WMH volumes were skewly distributed and we used log WMH, in addition to medians (range), also given as geometric mean (SD) in order to compare with other studies. Several controls had no WMH, therefore we used log WMH (0 + 0.0001) for all controls. All data were analyzed by using statistical package for social sciences (SPSS) version 14 for windows, and a P value less than 0.05 was considered as statistically significant.

## Results

### Patient characteristics and brain structure

Clinical characteristics of all patients and cerebral MRI measurements in all patients, in patients with or without CAD and age and sex matched controls, respectively, are summarized in Table 1. Of note, WMH were higher and BPF was lower in our patients, and in particular patients with CAD, compared to age and sex matched controls, however not when adjusting for conventional risk factors.

**Table 1:** The clinical characteristics and cerebral measurements of all patients and patients with or without asymptomatic coronary artery disease (CAD) defined as abnormal myocardial perfusion imaging and/or stenosis on coronary angiography, and age and sex matched controls

	**All patients**	**Controls**	**p-values**^**1**^	**Patients without CAD**	**Patients with. CAD**	**p-values**^**2**^
	**(n = 20)**	**(n = 26)**		**(n = 10))**	**(n = 10))**	
Sex no. (male%)	17 (76)	21 (81)	0.70	8 (80)	9 (90)	0.53
Age, years	57 (10)	52 (15)	0.28	51 (9)	63 (7)	0.005
Duration of diabetes, years	12 (6)			9 (6)	15 (7)	0.036
BMI, kg/m^2^	31.9 (4.3)			31.5 (4.3)	32.2 (4.6)	0.69
HbA_1c_ , mmol/mol , (%)	63 (7.9)			53 (7.8)	64 (8.1)	0.66
Urinary albumin excretion rate, mg/24h^a^	103 (3 – 1263)			118 (42–618)	95 (3 – 1263)	0.37
P-creatinine, μmol/l	78 (20)			69 (17)	88 (18)	0.025
Systolic blood pressure, mmHg	133 (17)			127 ± 15	138 (18)	0.20
Total cholesterol, mmol/l	3.7 (0.9)			3.8 ± 1.1	3.5 (0.7)	0.43
Vibratory perception threshold mV – mean of both sides	31 (15)			22 (12)	40 (13)	0.004
Heart rate variation during deep breathing, bpm^a^	8 (2–29)			11 (5–29)	5 (2–11)	0.004
Retinopathy no. (%)	11 (55)			4 (40)	7 (70)	0.18
Oral antidiabetic medication no. (%)	18 (90)			9 (90)	90 (90)	1.0
Insulin treatment no. (%)	14 (70)			6 (60)	8 (80)	0.33
RAAS blockade no. (%)	20 (100)			10 (100)	10 (100)	1.0
Statin therapy no. (%)	20 (100)			10 (100)	10 (100)	1.0
Aspirin therapy no. (%)	20 (100)			10 (100)	10 (100)	1.0
Beta-blocker therapy no. (%)	2 (10)			0 (0)	2 (20)	0.14
Calcium channel blockers no. (%)	7 (35)			1 (10)	6 (60)	0.015
Use of diuretics no. (%)	11 (55)			5 (50)	6 (60)	0.65
Current smoker no. (%)	5 (25)			3 (30)	2 (20)	0.60
Carotid intima-media thickness, mm	0.73 (0.14)			0.70 (0.13)	0.77 (0.14)	0.34
NT-proBNP, ng/l^a^	23.9 (5.1-357,6)			7.6 (5.1-29.1)	125.4 (15.8-357.6)	nr
MMSE^a^	30 (25–30)			30 (28–30)	28 (25–30)	0.035
**Cerebral MRI measurements**						
White matter hyperintensities, ml^b^	0.79 (4.89)	0.002 (139)	0.0001	0.34 (2.75)	1.85 (5.17)	0.012
White matter hyperintensities, ml^a,b^	0.48 (0.29-2.92)	0 (0–1.40)	0.0001	0.42 (0.13-0.78)	2.46 (0.36-2.92)	0.012
Brain parenchymal fraction	0.77 (0.04)	0.82 (0.05)	0.001	0.79 (0.04)	0.74 (0.02)	0.002
Intracranial volume (ICV), ml	1443.4 (123.9)	1441.5 (285.7)	0.98	1451.0 (104.7)	1435.8 (146.0)	0.79
Grey matter volume per ICV,%	38.9 (2.7)	42.6 (3.2)	0.0001	40.2	37.6	0.029
White matter volume per ICV,%	37.6 (2.3)	39.1 (2.1)	0.027	39.0	36.3	0.006
Cortex volume per ICV,%	29.9 (2.3)	33.0 (2.7)	0.0001	31.0	28.8	0.033
Ventricular volume per ICV,%	3.4 (1.3)	2.5 (0.8)	0.002	2.9	4.2	0.024

Data are expressed as means (standard deviation [SD]) or medians (interquartile range) ^a^. Not relevant (nr).

WMH volumes ^b^ are both expressed as geometric mean (SD) values or medians (interquartile range) ^a^ in order to compare with other studies. Several controls had no WMH and therefore we used log WMH (0 + 0.0001) for all controls. The majority of controls had no signs of WMH (69%) but in contrast all patients had WMH.

P-values reflect comparison between patients and controls^1^, and patients with or without CAD^2^.

These comparisons were all non-significant when adjustment for age and sex.

Mini mental state examination (MMSE).

### P-NT-proBNP, WMH volume and BPF

**Figure 1:**
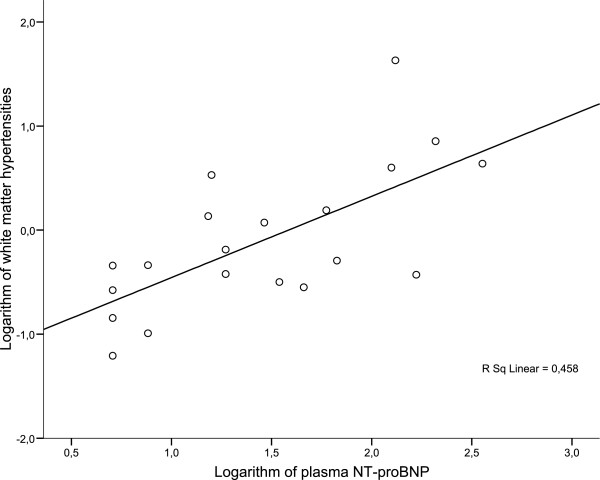
Univariate linear regression analysis with the logarithm of plasma NT-proBNP (ng/l) in relation to the logarithm of white matter hyperintensities in 20 patients with type 2 diabetes.

P-NT-proBNP was measured in all cases and distribution is shown in Table 1. A correlation in univariate linear regression analysis was found between P-NT-proBNP and WMH volumes (r = 0.68, p = 0.001, Figure 1). In an adjusted model that included age, sex, total cholesterol and systolic blood pressure, P-NT-proBNP remained predictive of WMH (r = 0.82, p = 0.05). Importantly, when all the other previously defined CV risk factors (known diabetes duration, total cholesterol, BMI, systolic blood pressure, HbA1c, presence of retinopathy, urinary albumin excretion rate, vibration threshold, heart rate variation, CIMT, LVEF, CCS or the presence of asymptomatic CAD) were included in the latter analysis instead of total cholesterol and systolic blood pressure in various combinations as previously defined, the association remained significant (all p < 0.05). Interestingly, when CIMT was included in addition to age, sex, cholesterol and systolic blood pressure, the significance and association between P-NT-proBNP and WMH increased (r = 0.94, p = 0.001). P-NT-proBNP was also associated with BPF in univariate linear regression analysis (r = 0.57, p = 0.009) but not after adjustment for sex and age (p = 0.23).

**Figure 2:**
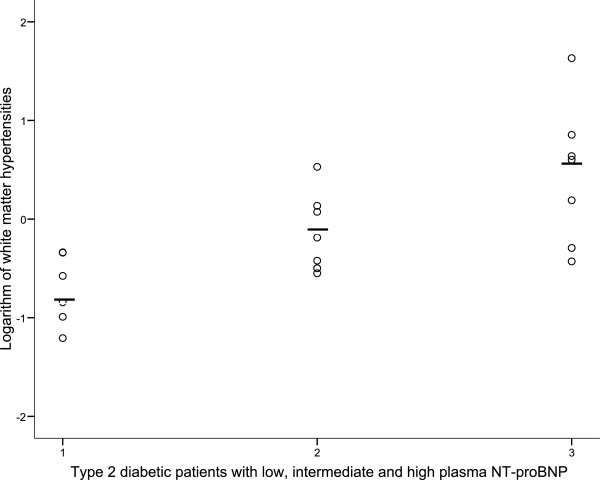
A scatter plot correlation, showing white matter hyperintensities in 20 patients with type 2 diabetes and with low, intermediate and high plasma NT-proBNP levels (tertile values), respectively, (horizontal bar is geometric mean among the groups, p = 0.003).

Patients were also divided into NT-proBNP tertiles (<14.7, 14.7-57.4, and >57.4 ng/l, respectively), and patients with high and intermediate P-NT-proBNP levels had higher WMH volume (geometric mean[SD]; (2.86[5.11] and 0.76[2.49], compared to patients with low P-NT-proBNP(0.20[2.28] , p = 0.003, Figure 2), and this was also significant when adjusting for age, sex and presence of CAD (p = 0.017).

## Discussion

### Principal findings

In twenty patients with type 2 diabetes with no history of stroke, P-NT-proBNP was strongly associated with volumes of white matter hyperintensities. Furthermore, white matter hyperintensities were higher and brain parenchyma fraction was lower in our patients, and in particular patients with CAD, compared to age and sex matched controls, however not when adjusting for conventional risk factors.

### Plasma NT-proBNP and white matter hypertensities

Our main finding that elevated P-NT-proBNP levels were associated with presence and severity of WMH has to our best knowledge not been described before. The association was independent of all tested CV risk factors and particular age and systolic blood pressure, which are considered the main predictors of WMH. Noteworthy, the association between P-NT-proBNP and WMH was strengthened and not attenuated by the addition of CV risk factors and in particular CIMT, and this may suggest that the cause is not related to an age systemic atherosclerosis but indeed organ heart and brain specific, therefore linking the heart with the brain. In humans the left ventricle is the main source of P-NT-proBNP with increased myocyte stretch or myocardial ischemia. It therefore seemed unlikely, based on physiology that elevated P-NT-proBNP from the heart *per se* directly promoted WMH or cerebrovascular disease in the present study. In contrast, heart disease such as heart failure or presence of atrial fibrillation that are known determinants of higher P-NT-proBNP levels and associated with increased risk of strokes, could be a common driver of both elevated P-NT-proBNP and WMH [7]. Our patients, however, were without acute illness and had normal ejection fraction without atrial fibrillation or valvular disease, and patients also had lower levels of P-NT-proBNP than seen in congestive heart failure [11]. Furthermore, we performed concomitant analyses with measures of subclinical heart disease, and the association between P-NT-proBNP and WMH remained significant after adjustment for LVEF, CCS and asymptomatic CAD. Along that line when patients with CAD were excluded, the association between P-NT-proBNP and WMH actually persisted. This is particular important, since the differences in P-NT-proBNP levels between patients with and without CAD were large and this could drive a false association between P-NT-proBNP and WMH. Of note, we only included patients with or without previously screen detected asymptomatic CAD in order to include patients with a wide risk profile. Accordingly, our present study suggests that the cause of the association between P-NT-proBNP and WMH is not explained by a common typical CV or heart disease driver, therefore possibly directly linking P-NT-proBNP and WMH. Along that line, recent studies have documented that plasma brain natiurectic peptide (BNP) itself increases the risk of stroke [16]. WMH are commonly found on MRI of elderly individuals with frequencies ranging from 11-21% at age 60 to 94% at age 82 [4]. Although often referred to as 'incidental', WMH have been associated with decline in cognitive function in independently living elderly populations and with a two-fold increased risk of dementia or stroke [4]. Furthermore, WMH are regarded as expressions of chronic hypoperfusion and small vessel cerebral disease. Small vessel cerebral disease is the most prevalent asymptomatic neurological disease, its incidence reported to be 6- to 10-fold that of symptomatic stroke [9,10]. Presence of WMH has been reported to predict a three-fold increased risk of stroke and two-fold increased risk of mortality in the general population [4]. In the recent Framingham Offspring Stroke Study, the presence of WMH predicted CV mortality and this was independent of risk factors and strokes or dementia [17]. Following the discussion of the mechanism underlying the relationship between P-NT-proBNP and WMH, the reverse could also be true; WMH induced elevated P-NT-proBNP levels through abnormal neuroendocrine signals to the cardiac ventricles and thereby functional asymptomatic heart failure [7]. Accordingly, the chronic cerebral hypoperfusion seen in patients with WMH could indirectly promote NT-proBNP realise from the heart. Our measurement of cardiac autonomic neuropathy (heart rate variability), however, did not change the association between P-NT-proBNP and WMH in the present study. Increased P-NT-proBNP levels could, however, also reflect other counter actions to the damages associated with WMH and accordingly mediated through other pathways. Along that line, other studies have suggested that BNP include several actions in addition to vasodilation and promotion of natriuresis and diuresis such as inhibition of the sympathetic nervous system and inhibition of several hormone systems, including the renin-angiotensin-aldosterone-system (RAAS), endothelins, cytokines, and vasopressin [18,19].

Finally, the correlation between P-NT-proBNP and WMH could also be brain to brain instead of heart to brain. Along that line, damage or hypoperfusion of the brain (WMH) might directly induce realise of NT-proBNP from the brain [20]. Brain natriuretic peptide (BNP) was actually first described in the porcine brain and accordingly later localized in the hypothalamus, thalamus, cerebellum, pons and cortex of the human brain [20]. Few studies have investigated BNP and NT-proBNP from the brain in disease. Specifically, in rats status epilepticus induced increase in plasma ANP (8), and occlusion of the middle cerebral artery stimulates BNP mRNA expression in rat brain tissues [21]. In humans, the BNP gene promoter region contains a hypoxia-inducible factor-1 binding site, which activates BNP expression [22], and in patients with subarachnoid hemorrhage plasma BNP concentrations were higher than controls. Some studies have also shown that P-NT-proBNP is increased in patients with acute ischemic stroke [6,7,23-25] but these studies may not have convincingly demonstrated that this association is independent from heart disease. In contrast, Tomati et al. examined all included patients with acute stroke with echocardiography in order to exclude the presence of heart disease and hereby investigate if BNP levels were elevated in patients with acute stroke, independently of heart disease. The study demonstrated that plasma BNP levels were indeed associated with the severity of cerebrovascular disease and particular volume of brain infarct size. One human CT study also recently suggested that the ischemic brain tissue *per se* release NT-proBNP into the circulation [26]. Importantly, all our patients were without acute illnesses; since acute disease such as stroke might be a condition that *per se* with sympathetic and catecholamines activity, cause stress of the heart and therefore BNP secretion. In contrast, our patients with CAD had higher WMH and lower BPF, however no longer after adjustment for age and sex (Table 1). Our study also demonstrated that WMH is higher and BPF lower in our patients compared to age-matched controls, where 69% had no signs of WMH.

### Clinical implications

All our patients had microalbuminuria and several studies have demonstrated that microalbuminuria identifies a subgroup of patients with increased morbidity and mortality from CV disease but recent studies also suggest that P-NT-proBNP can further stratify the group into patients with high or low risk for CV events [1,3,5]. Specifically, we have previously identified P-NT-proBNP as a powerful predictor of all cause and cardiovascular mortality that included strokes in patients with type 2 diabetes [5]. In that study, 80% of patients in the upper P-NT-proBNP tertile died, compared to 30% of patients in the lower tertile during 15 years of follow-up. The cause of poor prognosis in patients with elevated P-NT-proBNP is not known. We therefore performed the above described cross-sectional study of 200 patients with microalbuminuria and in this study, which examined patients for subclinical CAD and CV disease, we demonstrated no independent correlations between P-NT-proBNP and measurements of peripheral systolic blood pressure, CIMT, CCS, MPI, CAG or echo abnormalities [11-13]. P-NT-proBNP levels, however, have been shown by others to correlate with CIMT [27], CCS [28], MPI [29], CAG [30] and echo abnormalities [31] in other populations. Along that line, all our patients received intensive multifactorial intervention at Steno Diabetes Center for years before entering the study and this intervention aimed at CV prevention includes improved glycemic, lipid, and blood pressure control, as well as antithrombotic therapy and lifestyle modification according to international guidelines [32]. Intensive multifactorial treatment according to the Steno-2 trail reduces CV disease and mortality with 50% [2]. Of note, the medical treatment in our large cohort included statins (95% of patients), aspirin (92%), and RAAS-blocking agents (98%) and yielded mean total cholesterol levels of 3.7 mM, arterial blood pressures of 133/75 mmHg, and haemoglobinA1c levels of 53 mmol/mol (7.9%), respectively. We have therefore speculated that the intensive multifactorial medical treatment may be responsible for a weakening of the associations currently found between P-NT-proBNP levels and the previously described measurements of subclinical CAD and CV disease [11-13]. The Steno-2 trial showed that P-NT-proBNP levels might still hold prognostic capability despite multifactorial treatment [33]. Along that line, the association between P-NT-proBNP and WMH in the present study could suggest that the residual risk seen in these patients could be cerebrovascular and that this risk is revealed with increased levels of P-NT-proBNP. If P-NT-proBNP in general can be used as an independent biomarker in the detection of subclinical WMH or cerebrovascular disease is, however, not known. Along that line, our present study is small and included a relative selected population of type 2 diabetic patients. For an example, the intensive medical therapy prescribed in our patients but also the selection of high risk CV patients with microalbuminuria and/or asymptomatic CAD. Along that line, the confounders on P-NT-proBNP levels could be even more pronounced in complicated CV patients, and therefore more than we were able to adjust for.

Today, it is generally accepted that the P-NT-proBNP assay may be useful in the screening, diagnosis and perhaps monitoring of patients with heart failure. In the general population, BNP inversely correlates with hyperinsulinemia but in diabetic patients P-NT-proBNP levels are increased compared to controls, and in addition BNP levels are altered by several factors in stroke patients [34-36]. P-NT-proBNP is a prognostic marker of CV disease in several populations including diabetic patients, and perhaps also a diagnostic tool for LV dysfunction in diabetic patients [5,31]. As described above, recent studies have demonstrated that BNP might also be important in patients with stroke. Specifically, Saritas et al., showed that plasma BNP levels could serve as a measurement of the severity and clinical progression in stroke patients [37]. Furthermore, the Framingham Offspring Study demonstrated that BNP offered improvement in the accuracy of stroke risk [16]. This could also be true in diabetic patients but is not formally proven [5]. Finally, it is important to note that few studies have investigated if WMH or other cerebrovascular disease in these patients on intensive medical therapy could serve a CV preventive treatment target. In one study, more aggressive antihypertensive treatment was associated with reduced progression in WMH but if this reduction is associated with reduced risk is not known [38]. Accordingly, the clinical impact of the presence of WMH and the true meaning of NT-proBNP and WMH associations need to be investigated in larger and longitudinal studies.

### Strengths and limitations

Our detection of brain structure in the present study was performed with 3D MRI scan, which is a well established, recognized and accurate strong non-invasive modality for these measurements. The MRI is sensitive with a three-dimensional quantification and low covariability [39]. Most studies that include MRI measurements in patients with diabetes are from subpopulations [40] and fewer studies report volumetric measurements as in the present study [41,42]. Although our study only included twenty patients with diabetes, we demonstrated a broad variation of WMH and brain volumes among our patients that were selected among 200 patients with or without asymptomatic CAD in order to include patients with a wide-ranging CV risk profile. The several multivariate analyses we performed as described in various combination of 2–3 CV risk factors in addition to age and sex, due to the relative low sample size, should obviously be interpreted with caution and we did not test for the multiple testing in these analyses.

## Conclusion

We demonstrated for the first time that plasma NT-proBNP was independently associated with white matter hyperintensities in asymptomatic type 2 diabetec patients with no history if stroke. The association was strengthened and not attenuated with the addition of CV risk factors and particular carotid artery intima media thickness, suggesting a linkage between heart and brain disease. We were however, not able to demonstrate a common driver of both P-NT-proBNP and white matter hypertensities. Finally, white matter hyperintensities were higher and brain parenchyma fraction was lower in our patients, and in particular patients with CAD, compared to age and sex matched controls, however not when adjusting for conventional risk factors.

## Abbreviations

CCS: Agaston coronary calcium score; BPF: Brain parenchymal fraction; CV: Cardiovascular; CIMT: Carotid artery intima media thickness; CAG: Coronary angiography; CAD: Coronary artery disease; LVEF: Left ventricular ejection fraction; MRI: Magnetic resonance imaging; MMSE: Mini Mental Status Examination; MPI: Myocardial imaging; P-NT-proBNP: N-terminal-proBNP; WMH: White matter hyperintensities.

## Competing interests

Dr. Rossing reports having received lecture fees from Novartis and Boehringer Ingelheim, and research grant from Novartis, has served as a consultant for Merck, and having equity interest in NovoNordisk. Dr. Parving reports having served as a consultant for Novartis, Merck, Pfizer and Sanofi-Aventis, having equity interest in Merck and NovoNordisk and having received lecture fees from Novartis, Merck, Pfizer and Sanofi-Aventis. Dr. Parving has received grant support from Novartis, AstraZeneca and Sanofi-Aventis.

## Author contributions

H.R: researched data, contributed to discussion, wrote manuscript. E.G., A.S, P.Å, T.R, K.W, PK.J, P.R, H-H.P: researched data, contributed to discussion, reviewed/edited manuscript. All authors read and approved the final manuscript.
